# Intraoperatively diagnosed spontaneous rupture of a subcapsular liver hematoma with incomplete hemolysis, elevated liver enzymes, low platelets (HELLP) syndrome: A case report and literature review

**DOI:** 10.1097/MD.0000000000044186

**Published:** 2025-08-29

**Authors:** Yanming Kang, Dongxu Chen, Xiang Li, Zhen Xia, Xiaoqin Jiang

**Affiliations:** a Department of Anaesthesiology, West China Second Hospital, Sichuan University, Chengdu, China; b Key Laboratory of Birth Defects and Related Diseases of Women and Children (Sichuan University), Ministry of Education, Chengdu, China; c West China School of Medicine, Sichuan University, Chengdu, China; d Department of Anesthesiology, Chengdu Hi-Tech Zone Hospital for Women and Children, Chengdu, China.

**Keywords:** HELLP syndrome, laparoscopic surgery, preeclampsia, pregnancy, subcapsular liver hematoma

## Abstract

**Rationale:**

Subcapsular liver hematoma (SLH) during pregnancy is a rare but potentially life-threatening complication, often associated with hypertensive disorders such as preeclampsia and hemolysis, elevated liver enzymes, low platelets (HELLP) syndrome.

**Patient concerns:**

We report the case of a 37-year-old primipara who presented at 39 weeks of gestation with severe preeclampsia (blood pressure, 166/106 mm Hg; proteinuria, 2+). Notably, her prenatal course was marked by normal blood pressure and 3 negative urine protein tests.

**Diagnoses:**

The patient was diagnosed with incomplete HELLP syndrome and a ruptured SLH. This intraoperative diagnosis was confirmed by the identification of 800 mL of noncoagulated blood and a 4 × 2 cm left hepatic lobe subcapsular hematoma with semiactive bleeding.

**Interventions:**

Laparoscopic hematoma evacuation and hemostasis were successfully performed.

**Outcomes:**

The patient had a stable postoperative recovery. A systematic review of 99 cases from 71 studies was conducted, which documented HELLP syndrome in 78 cases, with maternal and fetal mortality rates of 13.1% and 33.3%, respectively. Notably, 88.8% of patients presented with pain, predominantly right upper quadrant (46%), whereas our case exhibited atypical left-sided pain.

**Lessons:**

This case, along with a literature review, underscores the importance of vigilance for SLH in hypertensive pregnancies, even in the absence of classic HELLP criteria, and advocates for prompt imaging, individualized treatment, and multidisciplinary coordination to optimize outcomes.

## 1. Introduction

Subcapsular liver hematoma (SLH) during pregnancy is a rare but potentially life-threatening complication, primarily associated with preeclampsia, eclampsia, or hemolysis elevated liver enzymes and low platelet count (HELLP) syndrome.^[1]^ The Tennessee Classification System diagnostic criteria for HELLP are hemolysis, characteristic peripheral blood smear, serum lactate dehydrogenase (LDH) ≥ 600 U/L, total bilirubin ≥1.2 mg/dL; elevated liver enzymes, defined as aspartate aminotransferase (AST) ≥ 70 U/L; and low platelet count defined as <100 × 10^9^/L.^[2]^ Partial or incomplete HELLP syndrome consists of only 1 or 2 of the triad,^[2–5]^ which further complicates diagnosis and management. Preeclampsia affects approximately 4.6% of pregnancies.^[6]^ The HELLP syndrome occurs in 0.5% to 0.9% of all pregnancies and up to 10% to 20% of cases with severe preeclampsia.^[4]^ SLH is reported in 0.9% to 2% of pregnancies complicated with preeclampsia or HELLP syndrome.^[4,7]^ A literature review^[8]^ indicates that maternal and fetal mortality rates associated with subcapsular liver hematoma were first reported to be as high as 59% and 62%. However, with advances in early diagnosis and management, recent studies indicate 10% to 15% for maternal mortality and 41% for perinatal mortality.^[9,10]^ These outcomes depend on the rupture of SLH, the timing of diagnosis, and the availability of therapeutic interventions.

We reported a case of ruptured SLH diagnosed intraoperatively associated with incomplete HELLP syndrome, which was successfully managed through laparoscopic surgery. This case, alongside a systematic literature review, aims to enhance understanding of the SLH outcomes, provide preliminary experience in rescue techniques, and guide therapeutic decision-making.

## 2. Case presentation

A 37-year-old primipara with a history of uterine fibroid resection, subclinical hypothyroidism, hyperlipidemia, and a high-risk pregnancy (advanced maternal age, multiple uterine surgeries, in vitro fertilization and embryo transfer, G6P0 + 5 weeks) was admitted at 39 weeks gestation for elevated blood pressure (166/106 mm Hg). Routine prenatal visits had shown normal blood pressure and 3 negative urine protein tests. No significant abnormalities were observed in blood pressure, laboratory parameters, or urine protein levels throughout the pregnancy (Table 1). On admission, laboratory findings revealed hemoglobin 147 g/L, platelets 97.0 × 10^9^/L, albumin 24.7 g/L, and urine protein 2+. Ultrasound showed minimal pleural effusion but no hepatic, pancreatic, or splenic abnormalities (Fig. 3A). Physical examination revealed a fundal height of 34 cm, abdominal circumference of 108 cm, left occiput anterior fetal position, and normal fetal heart rate (150 bpm). The diagnosis was revised from gestational hypertension to severe preeclampsia, and due to a scarred uterus and laparoscopic cerclage, the patient opted for a cesarean section.

**Table 1:** Laboratory findings during pregnancy.

Investigation	First trimester	Second trimester	Third trimester
Drug therapy*	Aspirin 75 mg qd	Aspirin 75 mg qd	Aspirin 75 mg qd
Blood pressure (mm Hg)	118/70	117/72	119/80
Hemoglobin (g/L)	126	120	124
Platelets (×10^9^/L)	194	92	102
Leukocyte count (×10^9^/L)	10.3	8.7	7.4
ALT (U/L)	14	N/A	19
AST (U/L)	15	N/A	17
LDH (U/L)	134	N/A	143
Total bilirubin (µmol/L)	11.7	N/A	14.4
Uric acid (µmol/L)	252	N/A	418
Photostatin C (mg/L)	0.65	N/A	0.98
Creatinine (µmol/L)	43	N/A	50
Urinary protein	+−	−	−

Coagulation function shows no significant abnormalities during pregnancy.

ALT = alanine aminotransferase, AST = aspartate aminotransferase, LDH = lactate deshydrogenase, N/A = not available.

* Aspirin treatment from 13 + 6 weeks to 36 weeks.

**Figure 1.**
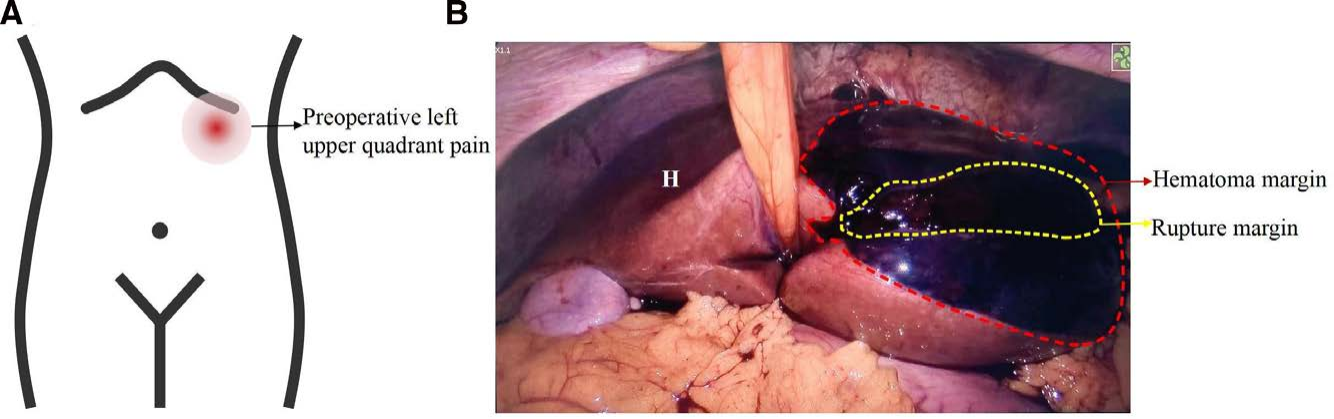
(A) Preoperative left upper quadrant pain: aggravated in the supine position and left lateral position (red dotted = tenderness area). (B) Laparoscopic view showing a 4 × 2 cm liver capsule rupture with semi-active bleeding in segment III. Red dashed line: hematoma margin; yellow dashed line: capsular rupture margin. H: hepatic surface.

**Figure 2.**
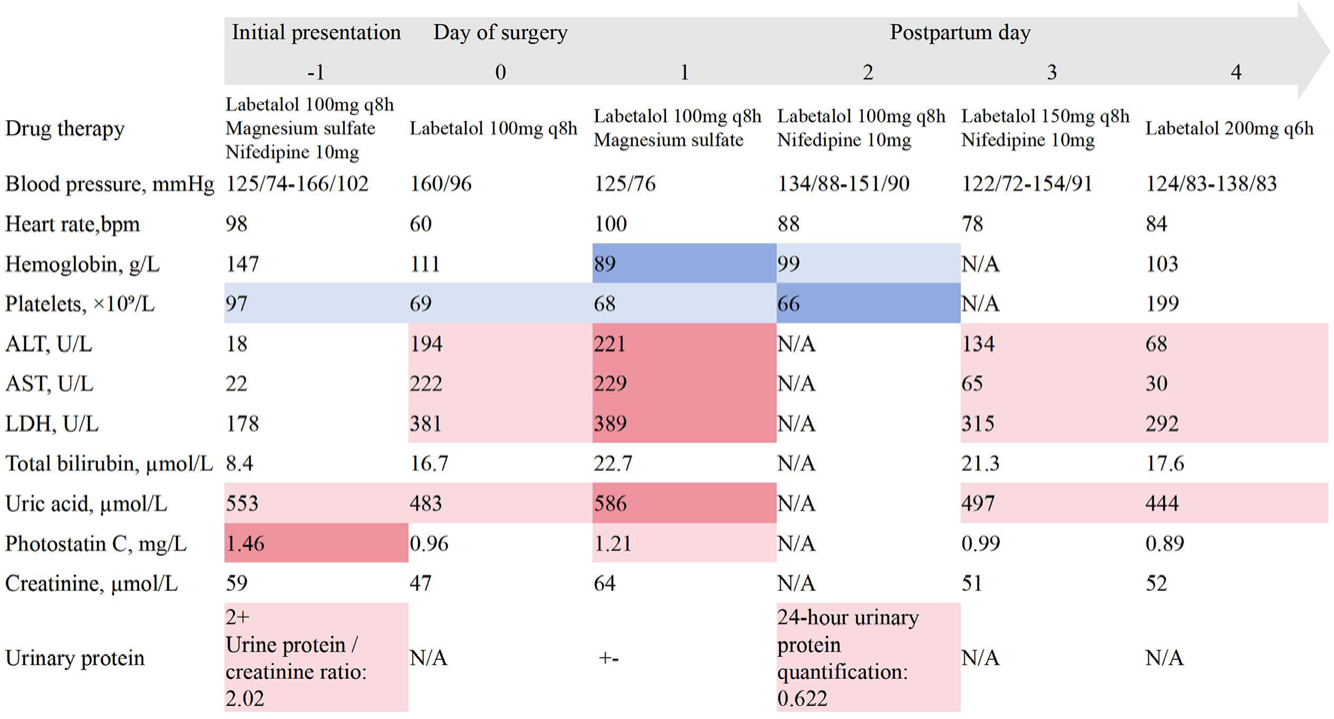
Perinatal examination results and treatment. Color coding: blue: values < lower normal limit (darker: greater reduction); red: values > the upper normal limit (darker: greater elevation); white: normal range. Coagulation parameters remained normal throughout (not shown). ALT = alanine aminotransferase, AST = aspartate aminotransferase, LDH = lactate dehydrogenase, N/A = not available.

**Figure 3.**
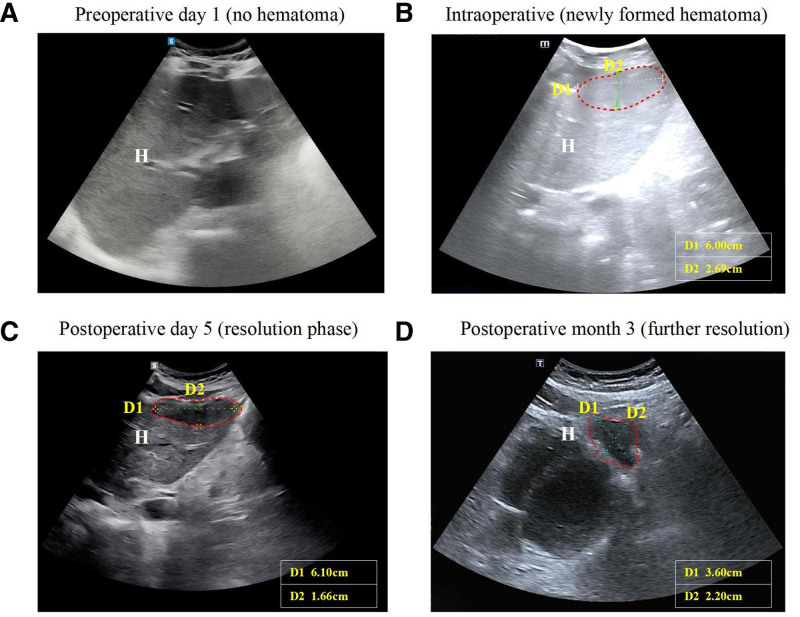
(A) Preoperative day 1: baseline scan showing normal hepatic parenchyma without hematoma. H: liver. (B) Intraoperative: newly formed hematoma. D1: 6.00 cm; D2: 2.69 cm; H: liver. (C) Postoperative day 5: hematoma in resolution phase. D1: 6.10 cm; D2: 1.66 cm; H: liver. (D) Postoperative month 3: hematoma showing further resolution with significant size reduction. D1: 3.60 cm; D2: 2.20 cm; H: liver. Red dashed line: hematoma margin; D1: longitudinal diameter; D2: transverse diameter.

On the following morning, she reported mild left upper quadrant pain without headaches, visual disturbances, chest pain, dyspnea, or edema. Vital signs remained stable, and physical examination revealed a soft abdomen with no significant tenderness or rebound pain. Uterine examination revealed normal tone without hypertonicity or contractions, effectively ruling out uterine rupture and preterm labor (critical considerations in this patient with prior uterine surgery [fibroid resection] presenting with acute abdominal pain). The fetal heart rate was normal. Before the spinal anesthesia puncture, her blood pressure was 128/92 mm Hg and her heart rate was 75 bpm. Due to worsening left upper abdominal pain in the supine and left lateral positions (Fig. 1A), the patient was positioned in a left lateral knee-chest posture. This position provided immediate pain relief, enabling successful spinal anesthesia through improved lumbar flexion and ultimately avoiding the need for general anesthesia during cesarean delivery. Upon opening the abdomen, approximately 800 mL of dark red non-coagulated blood was unexpectedly found. A live infant with an Apgar score of 10-10-10 was promptly delivered. Systematic pelvic exploration revealed no identifiable bleeding sources, raising concern for upper abdominal hemorrhage and prompting urgent intraoperative hepatobiliary consultation. The hepatobiliary surgeon palpated congestion and swelling of the left liver lobe through the limited Pfannenstiel exposure, raising suspicion of SLH (though capsule integrity was not visually confirmed).

Given the patient’s sustained hemodynamic stability, laparoscopy was selected for definitive diagnosis owing to its minimally invasive nature and superior visualization. Following completion of the cesarean delivery, laparoscopic exploration was performed under general anesthesia. Laparoscopic examination revealed approximately 100 mL of blood in the splenic fossa, with a hematoma covering the diaphragm. A SLH measuring 4 × 2 cm with semi-active bleeding was observed in segment III (Fig. 1B). Intraoperative ultrasound was performed to assess the extent and depth of the hematoma, which guided the surgical decision between hematoma evacuation and liver lobectomy. The ultrasound revealed a hematoma with a maximum depth of approximately 3 cm (Fig. 3B). The surgeon controlled active bleeding from the liver surface capsule and removed the hematoma using Bicaval forceps, coagulated the wound, covered it with hemostatic gauze, followed by the placement of a drain. After SLH diagnosis, the intraoperative laboratory profile confirmed incomplete HELLP syndrome: thrombocytopenia (platelets 69 < 100 × 10^9^/L) and AST 222 ≥ 70 U/L satisfied Tennessee criteria. The patient remained hemodynamically stable with an intake of 2000 mL crystalloid, 500 mL colloid, 400 mL blood loss, and 900 mL intra-abdominal bleeding. Postoperative management included antihypertensive, hepatoprotective, and anticoagulant therapies (Fig. 2). Hemoglobin and platelet levels declined postoperatively, reaching their lowest values on day 1 (hemoglobin 89 g/L; platelets 68 × 10^9^/L). Peak biochemical markers on postoperative day 1 included: alanine aminotransferase (ALT) 221 U/L, AST 229 U/L, LDH 389 U/L, and uric acid at 586 µmol/L. Cystatin and urinary protein levels also increased. By postoperative day 2, biochemical markers began to normalize, and coagulation function remained stable throughout the perioperative period. The patient was discharged on postoperative day 5, with follow-up showing gradual hematoma absorption (Fig. 3C and D). The protocol for the case report was approved by the Ethics Committee of West China Second Hospital (No. 2024/427), and informed consent was obtained from the patient.

## 3. Summary of literature review

A systematic literature search was conducted following Preferred Reporting Items for Systematic reviews and Meta-Analyses guidelines on databases of PubMed, Web of Science, Embase, and Ovid to identify relevant studies published between 2000 and 2024. The Boolean query combined (“Pregnancy” OR “Gestation” OR “Obstetric”) AND (“Liver rupture” OR “Hepatic rupture” OR “Liver laceration” OR “Subcapsular hematoma”). Inclusion required confirmed SLH in hypertensive pregnancy with outcome data; exclusions comprised cases of liver rupture due to other causes and non-English publications. After duplicate removal (n = 94), 3 investigators (YK, XL, ZX) independently screened titles and abstracts, excluding 364 records. Full-text review of 137 articles excluded inaccessible (n = 49), non-case (n = 9), non-English (n = 8), and data-deficient (n = 15) studies, yielding 71 studies (99 cases) (Figure S1, Supplemental Digital Content, https://links.lww.com/MD/P815, Data Set S1, Supplemental Digital Content, https://links.lww.com/MD/P816).

The clinical characteristics of the 99 cases are summarized in Table 2. The median maternal age was 31.0 years (interquartile range [IQR]: 28.0–35.0). Hemodynamic monitoring showed the peak systolic blood pressure was 150.0 mm Hg (IQR: 117.0–170.0) and the peak diastolic blood pressure was 92.0 mm Hg (IQR: 69.8–105.0; n = 78). Laboratory findings revealed a significant reduction in hemoglobin (median 87.0 g/L, IQR: 68.8–100.0) and platelet count (median 58.5 × 10^9^/L; IQR: 38.5–97.5). Hepatic function was markedly impaired, with peak levels of ALT 464.0 U/L, AST 547.0 U/L, total bilirubin 28.4 µmol/L, and LDH 1020.0 U/L exceeding normal limits.

**Table 2:** Characteristics of 99 pregnant women with hepatic rupture/subcapsular hematoma.

	Not reported (n)	Value
Maternal age (yr), median (IQR)	2	31.0 (28.0–35.0)
Peak SBP (mm Hg), median (IQR)	20	150.0 (117.0–170.0)
Peak DBP (mm Hg), median (IQR)	21	92.0 (69.8–105.0)
Laboratory findings, median (IQR)		
Hb nadir (g/L)	38	87.0 (68.8–100.0)
PLT nadir (×10^9^ g/L)	19	58.5 (38.5–97.5)
ALT peak (U/L)	34	464.0 (177.0–1292.0)
AST peak (U/L)	24	547.0 (209.0–1680.0)
Total bilirubin peak (µmol/L)	73	28.4 (21.8–52.2)
LDH peak (U/L)	46	1020.0 (532.0–2740.5)
GA at presentation (weeks), n (%)	1	
14–26		9 (9.2)
27–36		47 (48.0)
37–41		38 (38.8)
Postpartum		4 (4.0)
Multiparous, n (%)	9	38 (42.2)
Delivery method, n (%)	2*	
Cesarean delivery		78 (80.4)
Vaginal delivery		18 (18.6)
Abortion		1 (1.0)
Hepatic rupture, n (%)	0	80 (80.8)
HELLP, n (%)	0	78 (78.8)
Diagnostic methods, n (%)	5	
Imaging examination		31 (33.0)
Intraoperative exploration		29 (30.9)
Intraoperative exploration and imaging examination		32 (34.0)
Autopsy		2 (2.1)
Treatment, n (%)	2	
Surgery		46 (47.4)
Conservative treatment		36 (37.1)
Surgery and embolization		5 (5.2)
Embolization only		5 (5.2)
Liver transplantation		3 (3.1)
Death		2 (2.0)
Maternal mortality, n (%)	0	13 (13.1)
Fetal mortality, n (%)	0	33 (33.3)
ICU admission, n (%)	5	76 (80.9)

Data presented as median (IQR) for non-normally distributed continuous variables and n (%) for categorical variables. Normality was assessed using Shapiro–Wilk tests (*P* < .05).

ALT = alanine aminotransferase, AST = aspartate aminotransferase, DBP = diastolic blood pressure, GA = gestational age, Hb = hemoglobin, HELLP = hemolysis, elevated liver enzymes, low platelets, ICU = intensive care unit, IQR = interquartile range, LDH = lactate dehydrogenase, PLT = platelets, SBP = systolic blood pressure.

* Two cases of fetal death with unspecified delivery methods, one of which was associated with maternal death.

Cesarean section was performed in 80.4% (78/97) of cases, while 19 patients had vaginal deliveries or miscarriages. Among the 99 cases, hepatic rupture occurred in 80, with 78 demonstrating concomitant HELLP syndrome. Diagnosis was established via imaging (33.0%), intraoperative exploration (30.9%), a combination of imaging and intraoperative findings (34.0%), and autopsy (2.1%). For treatment, 47.4% of cases underwent surgery, 37.1% received conservative treatment, 5.2% received both surgery and embolization, 5.2% had embolization only, 3.1% received liver transplantation, and 2.0% cases resulted in death. The maternal mortality rate was 13.1% (n = 13), and the fetal mortality rate was 33.3% (n = 33). Additionally, 80.9% of patients required intensive care unit admission. In our case, SLH was diagnosed through a combination of intraoperative laparoscopic exploration and ultrasound, followed by hematoma evacuation. This did not result in either maternal or fetal death, and the patient was monitored in the intensive care unit postoperatively.

As shown in Table 3, pain was the most common presenting symptom, affecting 88.8% (87/98) of patients. Right upper quadrant pain (41 cases) was predominant, followed by gastrointestinal symptoms (27 cases), including nausea, vomiting, diarrhea, and bloating. Other symptoms included chest pain, dyspnea, headache, confusion, fatigue, oliguria, and blurred vision. Hemodynamic instability was observed in 37 cases, comprising hypotension (n = 27), shock (n = 6), cardiac arrest (n = 3), and hypertension (n = 1). Notably, one postpartum patient died suddenly without prior symptoms, and autopsy confirmed hepatic rupture. Our case presented with left upper quadrant pain, which is different from the 41 out of 98 reviewed cases where right upper quadrant pain was the predominant symptom. Additionally, the patient did not exhibit hemodynamic instability, which contrasts with the most common symptom seen in the majority of cases.

**Table 3:** Initial symptoms and signs of hepatic rupture/subcapsular hematoma in pregnant women (n = 98).

Category	Symptoms	n	Category	Signs	n
Pain* (n = 87)	Right upper abdominal pain	41	Hemodynamic	Hypotension	27
	Nonspecific abdominal pain†	23		Shock	6
	Upper abdominal pain	17		Cardiac arrest	3
	Shoulder/back pain	14		Hypertension	1
	Lower abdominal pain	2	Neurological	Eclamptic seizure	5
	Left upper abdominal pain	1		Loss of consciousness	3
Gastrointestinal	Nausea, vomiting, diarrhea, bloating	27	Hematologic	Anemia	3
Others	Chest pain, shortness of breath	10		Bleeding	3
	Headache	3	Abdominal	Tenderness	2
	Confusion	3	Hepatic	Elevated liver enzymes	1
	Fatigue	2	Renal	Hematuria	1
	Oliguria	1	Obstetric	Absence of fetal movement	1
	Blurred vision	1			

* The sum of pain subcategories exceeds the total number of patients with pain (n = 87) because some patients reported pain at multiple sites.

† Nonspecific abdominal pain is defined as documentation of “abdominal pain” without localization in medical records.

## 4. Discussion

Liver hematoma in pregnancy was first described in 1844,^[11]^ and remains a rare but serious condition. The pathophysiology is not fully understood, but preeclampsia and HELLP syndrome contribute significantly through vasospasm, coagulation abnormalities, and fibrin deposition in hepatic capillaries, leading to necrosis, thrombosis, and eventual hemorrhage.^[12,13]^ Diagnosing spontaneous SLH is challenging, especially in the absence of trauma, anticoagulant use, or underlying liver disease (e.g., hepatocellular adenoma^[14]^).

In our systematic review cohort (n = 99), HELLP syndrome was documented in 78 cases, aligning with a previous report (39/49, 80%),^[10]^ suggesting a strong association between SLH and HELLP syndrome. However, Chen et al^[10]^ reported a case of a ruptured massive hepatic subcapsular hematoma occurring during emergency cesarean section in a patient with preeclampsia but without HELLP syndrome. Similarly, in our case, SLH was associated with incomplete HELLP syndrome rather than the full syndrome. These findings underscore the importance of maintaining vigilance for SLH not only in classic HELLP syndrome but also in incomplete forms and even in the broader context of hypertensive disorders of pregnancy. SLH in pregnancy can present with varied clinical manifestations. Alarm signs include a sudden onset of abdominal pain in the right hypochondrium, irradiated to the right shoulder, arterial hypertension, and clinical data of low heart flow-shock condition.^[15]^ However, in our case, the clinical presentation involved left upper quadrant pain, which contrasts with the more commonly reported right upper quadrant pain in the literature. Pathophysiological insight suggests that this variability in symptoms is indicative of diffuse endothelial injury rather than isolated vulnerability of the right liver lobe. The presentation of left upper quadrant pain increases the diagnostic challenge, as it complicates differentiation from other acute abdominal conditions. These findings underscore the clinical significance of recognizing that any upper abdominal pain in hypertensive pregnancies warrants immediate hepatic ultrasound, regardless of the side of pain. This approach ensures timely identification and management of SLH, even in atypical presentations.

The differential diagnosis of SLH during pregnancy is extensive, encompassing both obstetric conditions, such as acute fatty liver of pregnancy, placental abruption with disseminated intravascular coagulation, uterine rupture, and idiopathic thrombocytopenia,^[1]^ as well as non-obstetric conditions such as peptic ulcer disease and hepatobiliary pathologies.^[8]^ This broad range of potential diagnoses presents a significant challenge in the differential diagnosis of abdominal pain during pregnancy. Diagnostic challenges are further highlighted by the incidental discovery of the hematoma during cesarean section. Notably, the limited exposure from a Pfannenstiel incision can restrict access to the upper abdomen, making it more difficult to detect SLHs, especially in patients with stable hemodynamics and no significant pain after anesthesia. Early suspicion and consideration of a midline vertical incision may be crucial for better abdominal exploration and confirmation of the hematoma. Early imaging is critical in confirming SLH. Ultrasound is preferred for hemodynamically unstable patients and intraoperative settings, while CT scanning is more sensitive for detailed assessment.^[16]^ Laboratory parameters of ALT, AST, LDH, and serum uric acid if elevated have been shown to predict a risk of more than 75% serious maternal morbidity in patients with pregnancy-induced hypertension.^[17]^

Treatment must be individualized based on hemodynamic stability, hematoma size, and bleeding activity, which primarily involves conservative and surgical approaches.^[16]^ For hemodynamically stable patients with an intact capsule, conservative treatment may be considered.^[18]^ Our case demonstrates the feasibility and safety of laparoscopic exploration in hemodynamically stable patients with suspected SLH. Laparoscopy offers several advantages over open surgery, including magnified visualization of the hepatic surface for precise hematoma assessment, reduced intraoperative blood loss, and decreased postoperative pain, leading to faster recovery and earlier maternal-neonatal bonding. However, if hemodynamic instability is present, urgent angiography and hepatic artery embolization are necessary,^[19,20]^ with/without surgical intervention including abdominal packing, suturing of liver parenchyma, or partial liver resection. Additionally, liver transplantation is regarded as a life-saving option for patients with acute hepatic failure.^[21,22]^ The deaths probably occurred due to ruptured SLH, primarily attributable to complications such as disseminated intravascular coagulation,^[23]^ hepatic infarction,^[15]^ acute respiratory distress syndrome, and renal failure.^[24,25]^ Therefore, it is crucial to identify SLH promptly to mitigate the mortality risk associated with hematoma rupture. Avoiding minor trauma, including excessive abdominal palpation, uterine contractions, and vomiting, is crucial to prevent hematoma expansion and rupture. Consequently, multidisciplinary coordination involving hepatic, obstetric, anesthesiology, and radiological expertise among other relevant specialties remains essential, as emphasized in complex obstetric care.^[26]^

We acknowledge several limitations in this case and the literature review. First, this study is based on a single-center experience, which may not fully reflect broader clinical practice. Second, the absence of the patient’s perspective limits the comprehensive understanding of the postoperative recovery experience. Additionally, this literature review is retrospective with the possibility of overlooked reports despite an extensive search, as well as the exclusion of case reports published in languages other than English.

## 5. Conclusions

This case and literature review emphasize the critical need for vigilance when managing hypertensive disorders in pregnancy, particularly incomplete HELLP syndrome, as SLH may present atypically. Symptoms such as epigastric pain, pallor, and hypotension (before or after delivery) should raise suspicion for SLH. Early detection through heightened clinical suspicion and prompt imaging, combined with timely surgical intervention and multidisciplinary care, are essential to optimizing maternal and fetal outcomes.

## Author contributions

**Conceptualization:** Dongxu Chen.

**Data curation:** Xiang Li, Zhen Xia.

**Supervision:** Xiaoqin Jiang.

**Visualization:** Yanming Kang.

**Writing – original draft:** Yanming Kang.

**Writing – review & editing:** Dongxu Chen.

## Supplementary Material


