# Supplementary figures and images for: Intraoperatively diagnosed spontaneous rupture of a subcapsular liver hematoma with incomplete hemolysis, elevated liver enzymes, low platelets (HELLP) syndrome: A case report and literature review
Source: Medicine (Baltimore). 2025 Aug 29;104(35):e44186. doi: 10.1097/MD.0000000000044186 (PMC12401199; doi:10.1097/MD.0000000000044186)

Supplemental Figure 1. Flowchart


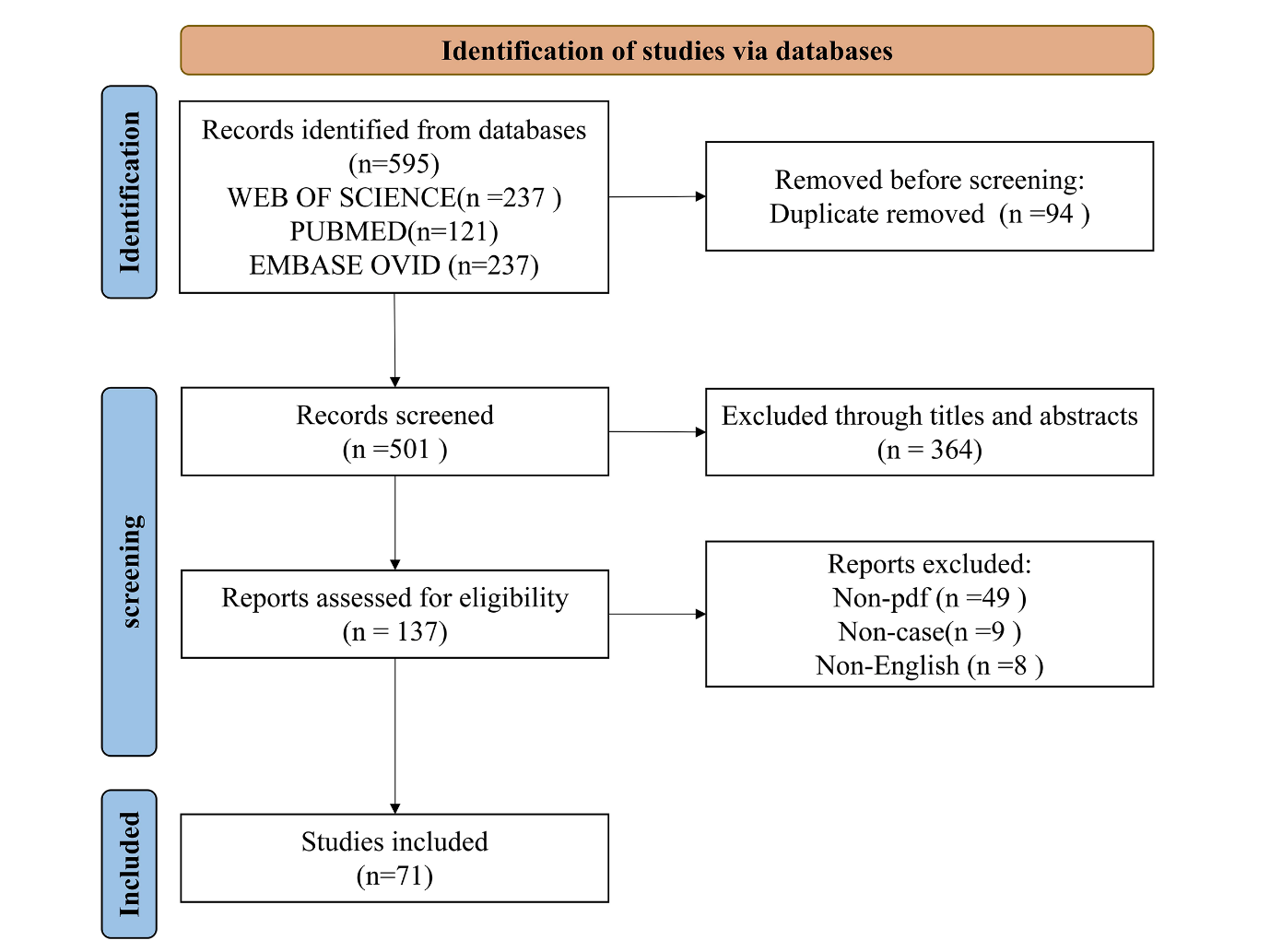

Supplement: Supplementary file 1 [file medi-104-e44186-s001.docx]
